# Rekindling Action Language: A Neuromodulatory Study on Parkinson’s Disease Patients

**DOI:** 10.3390/brainsci11070887

**Published:** 2021-07-01

**Authors:** Diana M. A. Suárez-García, Agustina Birba, Máximo Zimerman, Jesús A. Diazgranados, Pamela Lopes da Cunha, Agustín Ibáñez, Johan S. Grisales-Cárdenas, Juan Felipe Cardona, Adolfo M. García

**Affiliations:** 1 Facultad de Psicología, Universidad del Valle, Santiago de Cali 76001, Colombia; suarez.diana@correounivalle.edu.co (D.M.A.S.-G.); johan.sebastian.grisales@correounivalle.edu.co (J.S.G.-C.); 2 Cognitive Neuroscience Center (CNC), Universidad de San Andrés, Buenos Aires B1644BID, Argentina; agustina.birba@gmail.com (A.B.); mzimerman@ineco.org.ar (M.Z.); pamelopes@gmail.com (P.L.d.C.); agustin.ibanez@gbhi.org (A.I.); 3 National Scientific and Technical Research Council (CONICET), Buenos Aires C1033AAJ, Argentina; 4 Centro Médico de Atención Neurológica “Neurólogos de Occidente”, Santiago de Cali 76001, Colombia; dirgeneral@neurologosdeoccidente.com; 5 Agencia Nacional de Promoción Científica y Tecnológica (ANPCyT), Buenos Aires C1425FQD, Argentina; 6 Global Brain Health Institute (GBHI), University of California San Francisco (UCSF), San Francisco, CA 94143, USA; 7 Trinity College Dublin (TCD), D02R590 Dublin 2, Ireland; 8 Latin American Brain Health Institute (BrainLat), Universidad Adolfo Ibáñez, Santiago 8320000, Chile; 9 Faculty of Education, National University of Cuyo (UNCuyo), Mendoza M5502GKA, Argentina; 10 Departamento de Lingüística y Literatura, Facultad de Humanidades, Universidad de Santiago de Chile, Santiago 9170020, Chile

**Keywords:** Parkinson’s disease, action-verb processing, motor circuits, transcranial direct current stimulation, embodied cognition

## Abstract

Impairments of action semantics (a cognitive domain that critically engages motor brain networks) are pervasive in early Parkinson’s disease (PD). However, no study has examined whether action semantic skills in persons with this disease can be influenced by non-invasive neuromodulation. Here, we recruited 22 PD patients and performed a five-day randomized, blinded, sham-controlled study to assess whether anodal transcranial direct current stimulation (atDCS) over the primary motor cortex, combined with cognitive training, can boost action–concept processing. On day 1, participants completed a picture–word association (PWA) task involving action-verb and object-noun conditions. They were then randomly assigned to either an atDCS (*n* = 11, 2 mA for 20 m) or a sham tDCS (*n* = 11, 2 mA for 30 s) group and performed an online PWA practice over three days. On day 5, they repeated the initial protocol. Relative to sham tDCS, the atDCS group exhibited faster reaction times for action (as opposed to object) concepts in the post-stimulation test. This result was exclusive to the atDCS group and held irrespective of the subjects’ cognitive, executive, and motor skills, further attesting to its specificity. Our findings suggest that action-concept deficits in PD are distinctively grounded in motor networks and might be countered by direct neuromodulation of such circuits. Moreover, they provide new evidence for neurosemantic models and inform a thriving agenda in the embodied cognition framework.

## 1. Introduction

Parkinson’s disease (PD) is the second most prevalent neurodegenerative disorder [1] and the fastest growing source of disability [2] worldwide. Due to the disruption of frontostriatal motor circuits, patients manifest movement abnormalities [3,4], executive dysfunction [5], and, more particularly, deficits in action conceptualization [6,7,8]. The latter impairments are highly disruptive, as the ability to name, comprehend, and categorize actions is a cornerstone of adequate daily communication, interaction, and functionality [9,10,11]. However, no study has examined whether action semantic skills in persons with this disease can be modulated via non-invasive neuromodulation. Here we combined anodal transcranial direct current stimulation (atDCS) with cognitive training (CT) to explore whether neuromodulation of primary motor cortex (M1) activity boosts action–concept processing.

Early PD involves selective deficits in processing action concepts, i.e., verbal and pictorial stimuli evoking physical movement [6,7]. These impairments are directly tied to dopamine bioavailability [12,13], emerge irrespective of the patients’ executive and overall cognitive skills [14,15], and affect the production [13,16] and comprehension [17] of natural discourse. Moreover, selective or differential deficits for action categories in PD seem to be task-independent, as they have been observed in action-word naming [18], action-verb production [19], action-verb identification [20], and concept association [21] studies. At the neuronal level, action-concept alterations in PD seem to reflect abnormal M1 dynamics [22]. Indeed, in healthy persons, action concepts hinge critically on M1 activity [23,24,25,26,27] and functional connectivity among motor-preferential sites [28]. Thus, M1 neuromodulation in PD could impact action-concept processing.

This conjecture can be effectively tested through atDCS, a non-invasive neuromodulatory technique [29,30,31,32]. Despite some controversies [33] and mixed results [34,35], atDCS has been observed to modulate cortical excitability in and around the stimulated region [36], often leading to improved performance in putative functions [37,38]. In healthy individuals, atDCS of M1 boosts action-verb processing [39], action-word learning [40,41], and action-imagery benefits on motor outcomes [42,43,44]—but see Reference [27]. Conceivably, such effects partly reflect the impact of atDCS on motor circuits, as this intervention may increase blood flow in the left M1 [45] and corticostriatal and thalamocortical circuits [46] implicated in action-concept processing [47].

As regards PD, no study has examined the impact of tDCS on patients’ action-concept skills [48]. However, M1 atDCS in this population can ameliorate other motor-circuit dysfunctions, such as bradykinesia [49,50,51,52] and freezing episodes [53], as well as impairments of gait cadence [54], balance [55,56,57], motor performance, and functional mobility [58,59,60,61,62,63,64]. Suggestively, too, atDCS of other frontal regions in PD can increase the patients’ verbal fluency and fronto-posterior activity patterns [65,66]. Therefore, similar effects could be observed for action-concept processing upon M1 stimulation.

Moreover, this prediction can be tested for specificity by including an assessment of non-action concepts. In particular, object and object-noun stimuli are the gold-standard benchmark condition for action-concept processing in PD [6]. Whereas action semantic deficits have been proposed as candidate disease markers irrespective of severity [6,8,21], object-noun outcomes are highly variable across cohorts, ranging from full preservation [20] to moderate and marked deficits [67], depending on the patients’ cognitive status [68]. Accordingly, detection of selective action-concept advantages upon M1 stimulation would attest to the distinct link between motor-network integrity and motion-related stimuli in PD.

Against this background, we examined whether a three-day M1 atDCS intervention, supported by CT, can selectively boost action-concept processing in early PD. Patients were randomly assigned to either an atDCS or a sham group. Both before and after the stimulation protocol, they completed a picture–word association (PWA) task involving action-verb and object-noun conditions. Based on previous findings, we hypothesized that, unlike sham participants, patients in the atDCS group would exhibit selective enhancements of action-concept processing. Moreover, to test the functional specificity of the predicted effects, we explored whether they were influenced by cognitive, executive, and motor skills across groups. Shortly, this study aims to assess new therapeutic options for a key communicative deficit in PD.

## 2. Materials and Methods

### 2.1. Participants

The study comprised 22 non-demented, functionally independent Spanish-speaking PD patients (five females and 17 males) recruited at two neurological centers in Cali, Colombia. Given our design (see below), this sample size reaches a power of 0.96 (Supplementary Materials Section S1.1). All participants were right-handed, as confirmed with the Edinburgh Inventory [69], and had normal or corrected-to-normal vision. Clinical diagnosis of PD was made by an expert neurologist (J.A.D), in accordance with the United Kingdom PD Society Brain Bank criteria [70]. Motor impairments in all patients were assessed with section III of the Movement Disorder Society–sponsored revision of the Unified Parkinson’s Disease Rating Scale (MDS-UPDRS-III) [71] and the Hoehn and Yahr scale [72]. Functional skills were rated with the Barthel Index [73] and the Lawton and Brody Index [74], while depression symptoms were examined via the Geriatric Depression Scale (GDS) [75]. Moreover, the patients’ general cognitive state was evaluated with the Colombian validation of the Addenbrooke’s Cognitive Examination Revised (ACE-R) [76], which has proven sensitive to detect mild cognitive impairment in PD [77,78,79]. Executive functions were examined through the INECO Frontal Screening (IFS) battery [80], an instrument that robustly captures relevant deficits in PD [15,17,21,81,82]. All patients were taking antiparkinsonian medication, and they were evaluated during the “on” phase in both the pre- and the post-stimulation phases. None of them had other neurological disorders, previous neurosurgical procedures, major psychiatric conditions, history of substance abuse, or metallic implants or stimulators in their heads or hearts.

Patients were randomly assigned to either the anodal tDCS group (PD-atDCS, *n* = 11) or to the sham tDCS group (PD-stDCS, *n* = 11). No statistically significant differences were found between the groups in terms of sex, age, years of education, and years since diagnosis. They also had similar scores in tests of motor skills (MDS-UPDRS-III), symptom progression (Hoehn and Yahr scale), functionality level (Barthel Index and Lawton and Brody scale), depressive symptomatology (GDS), general cognitive status (ACE-R), and executive skills (IFS). For statistical details, see Table 1.

Before joining the study, all participants read and signed a consent form in agreement with the Declaration of Helsinki. The study was approved by the Ethics Committee of Universidad del Valle.

### 2.2. Experimental Task

We created a PWA task based on stimuli from a picture-naming task previously reported in PD research [68]. The PWA task comprised 80 trials, each composed of a black-and-white image and an accompanying word. Half the items belonged to the action-verb condition, and the remaining half corresponded to the object-noun condition. Each condition was composed of 20 congruent trials (e.g., the picture of a couple dancing with the Spanish verb meaning *dance*) and 20 incongruent trials (e.g., the picture of someone kneeling together with the Spanish word meaning *swim*). Items from the two conditions were matched for key properties of their pictures’ name agreement and their words’ frequency, age of acquisition, imageability, number of phonemes, and number of syllables (Supplementary Materials Section S2, Table S1). No word exceeded three syllables. Moreover, we ensured that the actual dominant name of the picture in the incongruent trials did not have marked phonological or semantic overlap with its accompanying incongruent word.

Stimulus motility/manipulability was established through two norming studies involving 34 university students. For the action-verb trials, participants were requested to rate how much movement of the limbs and torso was needed to perform the action represented by each picture, on a scale from 1 (minimal) to 100 (maximal). For object-noun trials, participants were asked to rate how graspable and manipulable each item was, from 1 (minimal) to 100 (maximal). Initially, 100 pictures of each category were pre-selected from Druks and Masterson [83] and Bates et al. [84], respectively. Inter-subjective variability was reduced through practice trials with pre-rated items. Once rated, stimuli with an average score below 30 were classified as having low motility/manipulability, and those with an average score above 60 were considered as having high motility/manipulability. Only 40 items were retained for each category, half the items comprising low motility/manipulability (actions: *M* = 18.14, *SD* = 6.96, range = 7.56–29.97; objects: *M* = 13.75, *SD* = 6.15, range = 4.60–29.90), and the other half comprising high motility/manipulability (actions: *M* = 76.56, *SD* = 14.65, range = 60–99.12, objects: *M* = 77.85, *SD* = 9.54, range = 61.85–93.82). This way, whereas all final action-verb items involved bodily movements and all object-noun items involved manipulable entities, both variables encompassed a substantial range of variability such that ensuing results would not be only valid for highly circumscribed subsets of items. 

Each trial began with a fixation cross (shown for a random period of 100–300 ms), followed by a two-element display composed of a picture and a word placed immediately below it. The picture–word dyad remained visible until the participant responded. Stimuli were presented in black color in the center of the screen against a white background. Sitting comfortably at a desk with a computer, participants were instructed to view each trial and press the right arrow to indicate ‘match’ or the left arrow to indicate “no match”. They were asked to perform the task as fast and accurately as possible. Each keystroke served to record the trial’s accuracy and response time (RT), while also triggering the following trial. The same sets of items were used in the pre-stimulation and post-stimulation sessions, guaranteeing pictorial and psycholinguistic comparability across conditions in each phase. Crucially, to minimize repetition or anticipation effects, blocks were presented with a different pseudorandomization in each phase, and all images with a congruent word in the pre-stimulation phase were accompanied by an incongruous word in the post-stimulation phase (and vice versa). As for the stimuli used for CT, these were chosen under qualitative agreement of neuropsychology and psycholinguistics experts (DMAS, AMG) to guarantee their categorical relevance and avoid repetitions with stimuli in the pre-stimulation and post-stimulation phases. Such CT stimuli were pseudorandomized under the same criteria used for the pre-stimulation and post-stimulation blocks. The action-verb and object-noun conditions were counterbalanced across participants and across sessions for each single participant. The use of separate blocks for action-verb and object-noun stimuli replicates a standard strategy in previous PD research [15,17,21,68,85,86] and other neurodegenerative diseases [86], maximizing comparability with relevant literature. Prior to the task, four practice trials (different from the 80 ones appearing in the task) were presented for familiarization purposes. Altogether, the task lasted approximately 10 min.

### 2.3. Experimental Protocol

**Figure 1:**
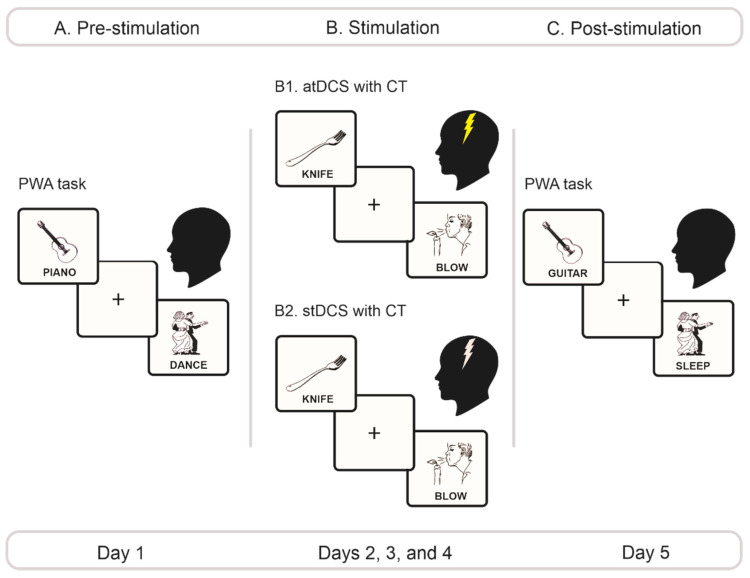
Experimental protocol. (**A**) Pre-stimulation phase. On day 1, we administered the PWA task, counterbalancing the order of the action-verb and the object-noun conditions across patients. (**B**) Stimulation phase. From days 2 through 4, depending on their group, patients underwent either atDCS (B1) or stDCS (B2) of the left M1 region during 20 min. Stimulation was applied with an intensity of 2 mA for the atDCS group and 0 mA for the stDCS group. Each day, during stimulation, they performed counterbalanced versions of the PWA task with stimulus sets that differed from those employed in the pre- and Ppost-stimulation phases. (**C**) Post-stimulation phase. On day 5, we repeated the same exact procedure of day 1, with stimuli from the PWA task presented in a new pseudorandomized order. PWA, picture–word association; atDCS, anodal transcranial direct brain stimulation; stDCS, sham transcranial direct brain stimulation; CT, cognitive training.

The study comprised three phases: (1) a pre-stimulation phase, (2) a stimulation phase, and (3) a post-stimulation phase (Figure 1). The entire protocol was performed over five consecutive days. All participants completed all sessions. The protocol’s length was strategic to minimize the possibility of dropouts. Previous studies have shown that three or fewer days of intervention with atDCS may be enough to induce significant effects on motoric [53,55,58,64,87], verbal [66], and otherwise cognitive [65,88,89] functions.

#### 2.3.1. Pre-Stimulation Phase

Participants performed the PWA task, following the procedure described in Section 2.2. The two conditions of the task (action-verb, object-noun) were counterbalanced across participants.

#### 2.3.2. Stimulation Phase

Based on previous studies, the anode electrode was placed on the left M1region [53,56,57,62,90], corresponding to electrode C3 on the international 10–20 system. The cathode was placed over the right dorsolateral prefrontal cortex, corresponding to electrode FP2. Direct electrical currents for anodal and sham stimulation were generated via a portable, stimulation device (Starstim, Neuroelectrics^®^, ISO 9001/13485 Barcelona, Spain) used in previous works [91,92,93]. Correct position of the electrodes was verified via the StimViewer software, which provides an integrated mathematical model of the spatial distribution of electrodes over the scalp according to the 10/10 international system. We used pairs of 25 cm^2^ circular electrodes covered with a saline solution to enhance signal transmission. Participants in the atDCS group received 20 min of stimulation with a current intensity of 2 mA. Subjects undergoing stDCS were only applied a direct current during 1 min, ramped up for 30 s at the beginning, and ramped down for the same time at the end of the stimulation session—a procedure demonstrated to warrant successful blinding [94]. A standardized questionnaire was administered by a neuropsychologist and a psychologist following each session to monitor for possible side effects such as headache, neck pain, scalp pain, tingling, itching, burning or burning sensation, redness of the skin, drowsiness, trouble concentrating, acute mood swings or any other side effect reported by the patients.

Over the three days of this phase, stimulation was performed online and accompanied by a CT protocol. Specifically, each day during stimulation, patients performed the PWA task with three stimulus sets different from the ones used in the pre- and post-stimulation phases. These versions of the task were identical in structure and number of items per condition. The two conditions were counterbalanced across participants in each stimulation day. All pictures in these sets were extracted from the same image banks used for the pre- and post-stimulation tasks (see Section 2.2). Although items in these three sets were not controlled for pictorial or lexical properties, they observed the following criteria: no word exceeded three syllables, the words in the incongruent trials were never the same used for congruent trials, and the actual dominant name of the picture in the incongruent trials did not have marked phonological or semantic overlap with its accompanying incongruent word. The same exact protocol was followed during the three days of the stimulation phase.

#### 2.3.3. Post-Stimulation Phase

The post-stimulation phase was performed on day 5 of the protocol. It was identical in structure and duration to the pre-stimulation phase. The only difference was that pictures of the PWA task which had been accompanied by congruent words in the pre-stimulation phase were now accompanied by incongruent words, and vice versa. The order of presentation of the object-noun and action-verb conditions was counterbalanced across subjects.

### 2.4. Data Analysis

#### Behavioral Data Analysis

As in previous embodied semantic experiments on PD and other motor disorders using verbal [17,68,95] and non-verbal [15,85,96] tasks, performance on the action and object conditions were subjected to separate between-group analyses. Differences in accuracy and RT outcomes were analyzed via 2 × 2 mixed effects ANOVAs, including a between-subjects factor (group: PD-atDCS and PD-stDCS) and a within-subjects factor (time point: pre-stimulation phase and post-stimulation phase). Furthermore, as in previous pre-/post-studies on action-language [97], we performed an additional analysis to control for potential baseline differences between groups and conditions. To this end, for each subject we subtracted the outcomes obtained in the post-stimulation phase from those in the pre-stimulation one. Then, for each condition separately, we compared the results of those subtractions between groups via unpaired two-tailed *t*-tests. As in previous research [21,82,98], RTs were analyzed only for correct trials after removing outliers at 2 *SD*s relative to each subject’s mean. Interaction effects were further scrutinized via Tukey’s HSD post-hoc tests. Furthermore, as in previous studies [15,17,68], we explored the functional specificity of significant effects by co-varying them for cognitive status (as captured by the ACE-R), executive skills (as assessed by the IFS battery), and motor skills (as tapped through the UPDRS-III). In all cases, alpha levels were set at *p* < 0.05. Effect sizes for main effects and interactions were calculated through partial eta squared (η_p_^2^), with cutoffs of >0.02, >0.13, and >0.26 for small, medium, and large sizes, respectively [99]. Effect sizes for pairwise comparisons in post-hoc analyses were performed with Cohen’s *d*, discriminating between small (0–0.20), medium (0.50–0.80), and large (>0.80) effect sizes [99]. All statistical analyses were carried out on Statistica 10.0 (http://www.statsoft.com/ (accessed on 1 May 2021).

## 3. Results

### 3.1. Behavioral Results

#### 3.1.1. Action-Verb Processing

Accuracy outcomes for action-verb processing revealed null effects of group [*F*_(1,20)_ = 0.27, *p* = 0.14, η_p_^2^ = 0.10] and time point [*F*_(1,20)_ = 0.25, *p* = 0.61, η_p_^2^ = 0.01]. The interaction between these two factors was also non-significant [*F*_(1,20)_ = 0.09, *p* = 0.76, η_p_^2^ < 0.01]; for details, see Supplementary Materials Section S3 (Table S2). Similarly, no between-group differences emerged in the subtraction analysis (Post-Minus-Pre accuracy analysis: *t*(20) = 0.30, *p* = 0.76, *d* = 0.12). For details, see Supplementary Materials Section S3 (Table S3).

After rejection of incorrect trials and outliers, the number of valid items for RT analysis did not significantly differ between groups [*F*_(1,20)_ = 0.08, *p* = 0.78, η_p_^2^ < 0.01]. Moreover, although more valid trials were found in the post- than in the pre-stimulation phase [*F*_(1,20)_ = 14.80, *p* < 0.01, η_p_^2^ = 0.42], the interaction between group and time point was not significant [*F*_(1,20)_ = 0.09, *p* = 0.77, η_p_^2^ < 0.01], attesting to the comparability of all critical conditions. For details, see Supplementary Materials Section S3 (Table S4). Bartlett tests revealed that the homoscedasticity assumption was met for this condition (Supplementary Materials Section S4).

**Figure 2:**
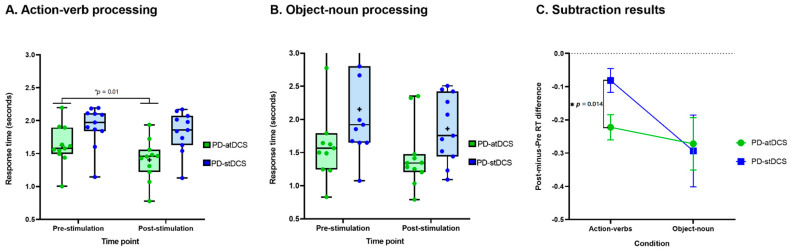
Behavioral results. (**A**) Response times for the action-verb condition revealed faster performance for the PD-atDCS than the PD-stDCS group. (**B**) Response times for the object-noun condition did not differ significantly between groups. (**C**) The subtraction analysis confirmed that the between-group difference proved consistent even when controlling for potential differences in baseline performance. The box and whisker plots in (**A**,**B**) show the distributions of RTs in the groups, with the boxes representing the inter-quartile range, the horizontal line within the boxes representing the median, and the points representing the individual mean RTs. The error bars in (**C**) represent standard error of the mean (SEM). PD-atDCS, Parkinson’s disease patients undergoing anodal transcranial direct current stimulation; PD-stDCS, Parkinson’s disease patients undergoing sham transcranial direct current stimulation.

Crucially, RT outcomes on the remaining action-verb trials revealed a main effect of group [*F*_(1,20)_ = 7.60, *p* = 0.01, η_p_^2^ = 0.27], with faster performance for PD-atDCS (*M* = 1.40 s, *SD* = 0.31) than PD-stDCS (*M* = 1.83 s, *SD* = 0.31) patients. The main effect of time point was also significant [*F*_(1,20)_ = 33.60, *p* < 0.01, η_p_^2^ = 0.62], with lower RTs after treatment. More importantly, a significant interaction emerged between these two factors [*F*_(1,20)_ = 7.22, *p* = 0.01, η_p_^2^ = 26]. A post hoc comparison via Tukey’s HSD test (MSE = 0.09, *df* = 21.64) revealed faster performance in the post-stimulation phase for the PD-atDCS group (*p* < 0.01), with no comparable effect in the PD-stDCS group (*p* = 0.15) (Figure 2A) For details, see Supplementary Materials Table S5. This effect remained significant after covariance for ACE-R, IFS, and UPDRSIII scores [*F*_(1,17)_ = 4.94, *p* = 0.04, η^2^ = 0.22]. Crucially, the subtraction analysis confirmed that the between-group difference proved consistent even when controlling for potential differences in baseline performance [Post-Minus-Pre RT analysis: *t*(20) = 2.68, *p* = 0.01, *d* = 1.21] (Figure 2C). For details, see Supplementary Materials Section S3 (Table S6). This effect also remained significant after co-varying for ACE-R, IFS, and UPDRS-III scores [*F*_(1,17)_ = 4.94, *p* = 0.04, η^2^ = 0.22].

#### 3.1.2. Object-Noun Processing

Accuracy outcomes for object-noun processing revealed null effects of group [*F*_(1,20)_ = 0.47, *p* = 0.50, η_p_^2^ = 0.02] and time point [*F*_(1,20)_ = 1.57, *p* = 0.22, η_p_^2^ = 0.07]. No significant interaction was found between these factors [*F*_(1,20)_ = 1.57, *p* = 0.22, η_p_^2^ = 0.07]. For details, see Supplementary Materials Section S3 (Table S2). Likewise, no between-group differences emerged in the subtraction analysis (Post-Minus-Pre accuracy analysis: *t*(20) = 1.25, *p* = 0.22, *d* = 0.53). For details, see Supplementary Materials Section S3 (Table S3).

Regarding RT, after rejection of incorrect trials and outliers, the number of remaining items did not differ significantly between groups [*F*_(1,20)_ = 1.05, *p* = 0.31, η_p_^2^ = 0.05] or time points [*F*_(1,20)_ = 0.03, *p* = 0.86, η_p_^2^ < 0.01]. Furthermore, there was not significant interactions between group and time point [*F*_(1,20)_ = 0.03, *p* = 0.86, η_p_^2^ < 0.01], confirming that the number of trials entering the RT analysis was similar for all critical comparisons. For details, see Supplementary Materials Section S3 (Table S4). Bartlett tests revealed that the homoscedasticity assumption was met for this condition (Supplementary Materials Section S4).

RT outcomes on the remaining object-noun trials revealed a null effect of group [*F*_(1,20)_ = 3.07, *p* = 0.09, η_p_^2^ = 0.13] and a significant effect of time point [*F*_(1,20)_ = 17.83, *p* < 0.01, η_p_^2^ = 0.47], revealing faster performance in the post-stimulation phase. The interaction between group and time point was not significant [*F*_(1,20)_ = 0.02, *p* = 0.87, η_p_^2^ > 0.01] (Figure 2B); for details, see Supplementary Materials Section S3 (Table S5). Moreover, neither was the difference between groups in the subtraction analysis [Post-Minus-Pre RT analysis: *t*(20) = 0.16, *p* = 0.87, *d* = 0.06]—Figure 2C. For details, see Supplementary Materials Section S3 (Table S6).

## 4. Discussion

This study examined whether neuromodulation of M1 activity can boost action-concept processing in PD. Selective enhancements were observed for action (as opposed to object) concepts in patients undergoing atDCS relative to those receiving sham stimulation. Moreover, these results were uninfluenced by the patients’ cognitive status, executive functions, and motor skills. Below we address these findings in turn.

Action-concept processing was significantly boosted only in the atDCS group, corroborating that this domain hinges on cortical motor activity in PD [22] and can be modulated upon M1 stimulation in different populations [27,40,41,42,43]. Importantly, no such effect was observed for object-noun outcomes. Note that similar dissociations between action and non-action categories upon motor-circuit disruptions in PD have been reported via lexical [20,68], picture-based [15,18,81,100], and discourse-level [16,17] tasks. In this sense, while action-verb deficits are systematic in this population [6,18,101,102], object-noun outcomes are markedly heterogeneous. Some samples show deficits in object-noun processing [17,68,103,104], whereas others exhibit preserved outcomes in this domain [20]. In particular, object-noun processing deficits seem to be associated with the patients’ cognitive state [15,68]. Our results extend these findings, showing that action-concept skills, relative to object-noun abilities, can be *selectively enhanced* upon modulation of motor regions in this population.

Moreover, this effect held irrespective of the patients’ cognitive status and executive skills. Previous reports indicate that action-concept deficits in PD are uninfluenced by domain-general impairment [6,14,15,17,21,68,81]. The same is true of action-concept effects induced through M1 atDCS [27] or ecological bodily training [97] in different populations. In line with these findings, our results indicate that action-concept gains following M1 stimulation in PD represent sui generis effects rather than secondary consequences of other unspecific cognitive changes. Likewise, the observed enhancements did not depend on the patients’ motor skills as tapped with the UPDRS-III. This aligns with previous works on PD showing that selective action-concept difficulties are not associated with their degree of motor impairment, as shown through picture-naming [68], lexical decision [20], word generation [19], and action fluency [105] tasks. Hence, present results suggest that not only action concept deficits, but also atDCS-based enhancements of this domain, might be potentially generalizable across PD patients independently of their degree of motor dysfunction.

These results bear clinical implications. Action-semantic alterations are paramount among the communicative profile of PD patients [13,16,17] and they constitute candidate cognitive markers for early PD [6,17]. Intake of levodopa or dopamine agonists seems to favor verbal action fluency [12,106] and action description in spontaneous speech [13], but such pharmacological interventions may have undesirable side effects. Promisingly, atDCS might circumvent some of such consequences and last for substantial time periods [36,57,107,108,109,110]. Accordingly, M1 neuromodulation might represent a complementary avenue to address action conceptualization disturbances in this population.

Note that the observed effect was driven by a combination of neuromodulation *and* task-specific CT. This corroborates the combined potential of both interventions to boost target domains [56,59,63,90,111,112]. Moreover, our results suggest that the observed action-concept improvements were not mainly driven by CT, as this intervention was present in sham participants, who exhibited no pre–post differences. Still, our design does not reveal whether M1 atDCS alone is sufficient to induce analogous effects, or if it acts by boosting proto-effects induced by CT. Suggestively, M1 neurostimulation, on its own, has been observed to selectively modulate action-concept processing in healthy subjects [27,40,113,114]. Future studies should explore whether the same is possible in PD cohorts.

Finally, from a theoretical viewpoint, our results support the “disrupted motor grounding hypothesis”, which claims that motor-network atrophy in PD should particularly disturb (higher-order) action-language processes [6]. More generally, these findings support the view that frontal motor circuits are critical for grounding action concepts [23,47,115,116], further suggesting a direct (partially causal) link between them. In this sense, our findings offer new empirical insights towards the development of fine-grained action-semantic models [117].

## 5. Conclusions

Our study shows that atDCS over M1, in combination with CT, can boost action-concept processing in early PD patients, irrespective of the patients’ cognitive, executive skills, and motor abilities. These results open new avenues to treat the pervasive action-semantic deficits observed in PD across experimental and naturalistic settings. Moreover, they afford new evidence to refine neurocognitive models of these domain and extend the promising neuromodulation agenda within embodied cognition research.

## 6. Limitations and Avenues for Further Research

This work presents some limitations that invite future studies. First, although our sample size was larger than that of other informative studies on PD [66,118], it was relatively small. Replications with larger groups would be desirable. Second, recruitment constraints prevented us from including a follow-up assessment to test the durability of the observed effects. Given that studies on other populations have shown that M1 stimulation can improve action-verb processing for up to four weeks [119], future research should test whether the reported enhancements are long- or short-lived. Third, the present study did not include neurophysiological measures, which hinders complementary neurocognitive interpretations regarding the detected effect. Thus, new implementations of our design should integrate online or offline brain measures, as in previous work [58,64,66,113,120,121]. Fourth, since our study aimed to assess the impact of M1 stimulation on action-concept processing in PD, the design did not require inclusion of a healthy control group. Still, future studies could replicate our work while incorporating such a sample to reveal whether the stimulation manages to abolish or merely attenuate the patients’ action-concept deficits. Moreover, this would allow for exploring whether the effects observed in PD patients manifest similarly or differentially in healthy participants [27]. Additionally, to further test the regional specificity of M1 neuromodulation on the observed results, future studies could complement our approach with stimulation of a control site, ideally accounting for possible propagation effects onto other cortical regions. Note that the UPDRS-III was included only in the pre-stimulation phase to establish the comparability of motor symptom severity between groups and rule out relevant confounds. We did not repeat this measure in the post-stimulation phase given that, unlike our fine-grained experimental task (based on objective time measures), UPDRS only captures gross aspects of motor behavior (through subjective impressions), which can hardly be robustly and discernibly modulated in a three-day stimulation protocol. Future studies could evaluate the impact of our protocol on motor behavior through appropriate tasks in both phases (e.g., motor learning or finger tapping paradigms). Finally, it would be useful to replicate our study with a crossover design, so as to better control for the potential impact of subtle subject-level factors on the observed results.

## Figures and Tables

**Table 1:** Demographic and clinical results.

	PD-atDCS (*n* = 11)	PD-stDCS (*n* = 11)	*p*-Value	Effect Size
Age	62.82 (7.49)	66.45 (5.69)	0.21 ^a^	0.53 ^c^
Years of education	11.45 (5.39)	8.09 (5.24)	0.15 ^a^	0.63 ^c^
Sex (F:M)	3:8	2:9	0.25 ^b^	---
Years since diagnosis	4.45 (0.30)	4.18 (3.84)	0.85 ^a^	0.09 ^c^
Hoehn and Yahr	1.09 (0.30)	1.09 (0.30)	1.00 ^a^	0.00 ^c^
MDS-UPDRS-III	18.64 (7.35)	19.45 (9.47)	0.82 ^a^	0.95 ^c^
GDS	0.45 (0.82)	0.91 (1.30)	0.33 ^a^	0.42 ^c^
Lawton and Brody	8.00 (0.00)	7.91 (0.30)	0.32 ^a^	0.59 ^c^
Barthel Index	100 (0.00)	98.64 (3.23)	0.17 ^a^	0.59 ^c^
ACE-R	92.00 (6.08)	87.27 (6.18)	0.08 ^a^	0.77 ^c^
IFS battery	19.64 (4.69)	19.09 (6.42)	0.82 ^a^	0.09 ^c^

Values are expressed as mean (*SD*), with the exception of sex. PD-atDCS, Parkinson’s disease patients undergoing anodal transcranial direct current stimulation; PD-stDCS, Parkinson’s disease patients undergoing sham transcranial direct current stimulation; F, female; M, male; MDS-UPDRS-III, Unified Parkinson’s Disease Rating Scale, part III; GDS, Geriatric Depression Scale; IFS, INECO Frontal Screening. ^a^ The *p*-values were calculated with two-tailed *t*-tests for independent samples. ^b^ The *p*-values were calculated by using chi-square test (*X*^2^). ^c^ Effect sizes were calculated with Cohen’s *d* metric.

## Data Availability

The data presented in this study are openly available online [122].

## Supplementary Materials

The following are available online at https://www.mdpi.com/article/10.3390/brainsci11070887/s1, Section S1: Sample size estimation, Section S2: Features of the stimuli in the picture-word association task, Table S1: Features of the stimuli in the experimental task, Section S3: Supplementary behavioral results, Table S2: Accuracy outcomes, Table S3: Post-minus-Pre accuracy, Table S4: Valid trials upon removal of incorrect trials and outliers for RT analysis, Table S5: RT outcomes, Table S6: Post-minus-Pre RTs, Section S4: Homoscedasticity tests.
