# Supplementary material for: Rekindling Action Language: A Neuromodulatory Study on Parkinson’s Disease Patients
Source: Brain Sci. 2021 Jul 1;11(7):887. doi: 10.3390/brainsci11070887 (PMC8301982; doi:10.3390/brainsci11070887)
Supplement: Supplementary file 1 [file brainsci-11-00887-s001.zip › brainsci-1277521-supplementary.pdf]

Supplementary data

1. Sample size estimation

To determine the sample size required for our analysis, we ran a power estimation on G*Power 3.1 [1]. Given our statistical design, based on a 2x2x2 mixed effects ANOVA, we considered three parameters. First, we established an alpha level of p = .05. Second, we considered a large effect size of $\eta 2$ = 0.20, based on previous studies that tested cognitive training and active tDCS on PD patients [2–6]. Finally, we established a power of 0.8. This analysis showed that a sample size of 8 is enough to reach the estimated effects. We considered an attrition rate of 30% increasing the targeted total sample size to 22. The actual sample size of our study (with each group featuring 11 participants) reaches a power of .96.

2. Features of the stimuli in the picture-word association task

Table S1. Features of the stimuli in the experimental task (data taken from Bocanegra et al., [7].

	Action-verb condition (n = 40)	Object-noun condition (n = 40)	p-value*
**Picture features**			
Name agreement[a]	93.38 (8.37)	93.30 (7.06)	.89
**Word features**			
Word frequency[b]	20.23 (24.94)	26.43 (23.88)	.06
Age of acquisition[c]	1.91 (1.01)	2.16 (.97)	.30
Imageability[d]	6.08 (.46)	6.18 (.53)	.09
Phonemes[d]	6.53 (1.65)	6.08 (1.62)	.20
Syllables[d]	2.48 (.68)	2.50 (.78)	.98

Data presented as mean (*SD*).
* p-values were calculated via Mann-Whitney U tests.
[a] Ratings obtained through a pilot study involving 34 participants.
[b] Data retrieved from the LEXESP database [8].
[c] Data retrieved from the International Picture-Naming Project (IPNP) corpus.
[d] Data retrieved from Valle-Arroyo [9]. When data was missing for specific stimuli, the values were obtained from a new group of 30 healthy volunteers.

3. Supplementary behavioral results

Table S2. Accuracy outcomes.

	PD-atDCS (n = 11)		PD-stDCS (n = 11)	
	Pre-stimulation	Post-stimulation	Pre-stimulation	Post-stimulation
Action-verbs	0.96 (0.03)	0.96 (0.04)	0.93 (0.06)	0.94 (0.05)
Object-nouns	0.97 (0.03)	0.97 (0.05)	0.97 (0.04)	0.95 (0.03)

Values are expressed, in percentage, as mean (*SD*). PD-atDCS: Parkinson's disease with anodal transcranial direct current stimulation; PD-stDCS: Parkinson's disease with sham transcranial direct current stimulation.

Table S3. Post-minus-Pre accuracy.

	PD-atDCS (n = 11)	PD-stDCS (n = 11)	p-value	Effect size
Action-verbs	.09 (1.97)	.36 (2.24)	.76[a]	.12[b]
Object-nouns	.00 (1.84)	-1(1.89)	.22[a]	.53[b]

Values are expressed, in percentage, as mean (*SD*). PD-atDCS: Parkinson's disease with anodal transcranial direct current stimulation; PD-stDCS: Parkinson's disease with sham transcranial direct current stimulation.

[a] p-values were calculated with unpaired two-tailed t-tests.

[b] Effect sizes were calculated with Cohen's d metric.

Table S4. Valid trials upon removal of incorrect trials and outliers for RT analysis.

	PD-atDCS (n=11)		PD-stDCS (n=11)	
	Pre-stimulation	Post-stimulation	Pre-stimulation	Post-stimulation
Actions-verbs	87.02 (4)	91.8 (2.97)	87.72 (4.3)	91.8 (4.1)
Object-nouns	89.07 (5.27)	89.07 (4.62)	90.45 (5.45)	90.9 (3.4)

Values are expressed, in percentage, as mean (*SD*). PD-atDCS: Parkinson's disease with anodal transcranial direct current stimulation; PD-stDCS: Parkinson's disease with sham transcranial direct current stimulation.

Table S5. RT outcomes.

	PD-atDCS (*n* = 11)		PD-stDCS (*n* = 11)	
	Pre-stimulation	Post-stimulation	Pre-stimulation	Post-stimulation
Action-verbs	1.62 (.31)	1.40 (.31)	1.91 (.31)	1.83 (.31)
Object-nouns	1.71 (.67)	1.44 (.48)	2.15 (.71)	1.86 (.51)

Values are expressed, in seconds, as mean (*SD*). PD-atDCS: Parkinson's disease with anodal transcranial direct current stimulation; PD-stDCS: Parkinson's disease with sham transcranial direct current stimulation.

Table S6. Post-minus-Pre RTs.

	PD-atDCS (*n* = 11)	PD-stDCS (*n* = 11)	*p*-value	Effect size
Action-verbs	.22 (.12)	.08 (.11)	.014[a]	1.21[b]
Object-nouns	.27 (.26)	.29 (.35)	.87[a]	.06[b]

Values are expressed, in seconds, as mean (*SD*). PD-atDCS: Parkinson's disease with anodal transcranial direct current stimulation; PD-stDCS: Parkinson's disease with sham transcranial direct current stimulation.
[a] *p*-values were calculated with unpaired two-tailed *t*-tests.
[b] Effect sizes were calculated with Cohen's *d* metric.

4. Homoscedasticity tests

Bartlett tests revealed that action-verb results met the homoscedasticity assumption in the between-group comparisons [pre-atDCS vs. post-atDCS: Bartlett's K-squared(1) = 0.004, $p = .95$; pre-stDCS vs. post-stDCS Bartlett's K-squared(1) < 0.001, $p = .99$; pre-atDCS vs. pre-stDCS Bartlett's K-squared(1) = 0.002, $p = .96$; post-atDCS vs. post-stDCS Bartlett's K-squared(1) < 0.001, $p = .98$]. The same was true for object-noun results [pre-atDCS vs. post-atDCS Bartlett's K-squared(1) = 0.98, $p = .32$; pre-stDCS vs. post-stDCS Bartlett's K-squared(1) = 0.98, $p = .32$; pre-atDCS vs. pre-stDCS Bartlett's K-squared(1) = 0.03, $p = .86$; post-atDCS vs. post-stDCS Bartlett's K-squared(1) = 0.03, $p = .87$]. This indicates that results in either category were not driven by their different variance.

Supplementary references

1. Faul F, Erdfelder E, Lang A, Buchner A. G*Power 3: a flexible statistical power analysis program for the social, behavioral, and biomedical sciences. Behav Res Methods. 2007;39(2):175–91.
2. Doruk D, Gray Z, Bravo GL, Pascual-Leone A, Fregni F. Effects of tDCS on executive function in Parkinson's disease. Neurosci Lett [Internet]. 2014;582:27–31. Available from: http://dx.doi.org/10.1016/j.neulet.2014.08.043
3. Biundo R, Weis L, Antonini A. tDCS effect on cognitive performance in Parkinson's disease. Movement Disorders. 2016.
4. Manenti R, Cotelli M, Cobelli C, Gobbi E, Brambilla M, Rusich D, et al. Transcranial direct current stimulation combined with cognitive training for the treatment of Parkinson Disease: A randomized, placebo-controlled study. Brain Stimulation. 2018;
5. París A, Saleta H, de la Cruz Crespo Maraver M, Silvestre E, Freixa M, Torrellas C, et al. Blind randomized controlled study of the efficacy of cognitive training in Parkinson's disease. Mov Disord. 2011;26(7):1251–8.
6. Naismith S, Mowszowski L, Diamond K, Lewis S. Improving memory in Parkinson's disease: A healthy brain ageing cognitive training program. Mov Disord. 2013 Jul;28(8):1097–103.
7. Bocanegra Y, García AM, Lopera F, Pineda D, Baena A, Ospina P, et al. Unspeakable motion: Selective action-verb impairments in Parkinson's disease patients without mild cognitive impairment. Brain Lang. 2017;
8. Sebastián-Gallés N, Carreiras M, Martí M, Cuetos F. LEXESP. Léxico informatizado del español. Barcelona: Universitat de Barcelona; 2000.
9. Valle-Arroyo F. Normas de imaginabilidad. Oviedo: Universidad de Oviedo, servicio de publicaciones; 1999.
